# Imaging Features of Pediatric Left Ventricular Noncompaction Cardiomyopathy in Echocardiography and Cardiovascular Magnetic Resonance

**DOI:** 10.3390/jcdd9030077

**Published:** 2022-03-05

**Authors:** Agata Paszkowska, Jędrzej Sarnecki, Alicja Mirecka-Rola, Monika Kowalczyk-Domagała, Łukasz Mazurkiewicz, Lidia Ziółkowska

**Affiliations:** 1 Department of Cardiology, The Children’s Memorial Health Institute, Al. Dzieci Polskich 20, 04-730 Warsaw, Poland; a.paszkowska@ipczd.pl (A.P.); a.mirecka-rola@ipczd.pl (A.M.-R.); m.kowalczyk-domagala@ipczd.pl (M.K.-D.); 2 Department of Diagnostic Imaging, The Children’s Memorial Health Institute, 04-730 Warsaw, Poland; j.sarnecki@ipczd.pl; 3 Cardiovascular Magnetic Resonance Unit, Department of Cardiomyopathies, National Institute of Cardiology, 04-682 Warsaw, Poland; lmazurkiewicz@ikard.pl

**Keywords:** left ventricular noncompaction, cardiomyopathy, imaging, echocardiography, cardiovascular magnetic resonance, children

## Abstract

*Background:* Left ventricular noncompaction (LVNC) is a distinct cardiomyopathy characterized by the presence of a two-layer myocardium with prominent trabeculation and deep intertrabecular recesses. The diagnosis of LVNC can be challenging because the diagnostic criteria are not uniform. The aim of our study was to evaluate echocardiographic and CMR findings in a group of children with isolated LVNC. *Methods:* From February 2008 to July 2021, pediatric patients under 18 years of age at the time of diagnosis with echocardiographic evidence of isolated LVNC were prospectively enrolled. The patients underwent echocardiography and contrast-enhanced cardiovascular magnetic resonance (CMR) with late gadolinium enhancement to assess myocardial noncompaction, ventricular size, and function. *Results:* A total of 34 patients, with a median age of 11.9 years, were recruited. The patients were followed prospectively for a median of 5.1 years. Of the 31 patients who met Jenni’s criteria in echocardiography, CMR was performed on 27 (79%). Further comprehensive analysis was performed in the group of 25 patients who met the echocardiographic and CMR criteria for LVNC. In echocardiography, the median NC/C ratio in systole was 2.60 and in diastole 3.40. In 25 out of 27 children (93%), LVNC was confirmed by CMR, according to Petersen’s criteria, with a median NC/C ratio of 3.27. *Conclusions:* (1) Echocardiography precisely identifies patients with LVNC. (2) Echocardiography is a good method for monitoring LV systolic function, but CMR is indicated for the precise assessment of LV remodeling and RV size and function, as well as for the detection of myocardial fibrosis.

## 1. Introduction

Left ventricular noncompaction (LVNC) is described as a distinct cardiomyopathy characterized by a two-layer myocardium with prominent trabeculation, deep intertrabecular recesses, and a thin compacted myocardial layer. LVNC was classified as a primary cardiomyopathy by the American Heart Association in 2006 [1] but remains unclassified by the European Society of Cardiology [2]. It typically involves the left ventricle, although involvement of the right ventricle (RV) has been reported [3]. LVNC can occur as an isolated or non-isolated phenotype. Non-isolated LVNC may be accompanied by congenital heart diseases or features of other cardiomyopathy or neuromuscular diseases. LVNC is a genetically determined myocardial disease, the third most common cardiomyopathy in the pediatric population (after dilated and hypertrophic cardiomyopathies). Molecular studies have confirmed the genetic etiology in approximately 40% of LVNC patients [4,5]. The clinical presentation is very heterogeneous, ranging from no symptoms to major events, such as heart failure, arrhythmias, thromboembolism, and sudden cardiac death [6,7,8].

The diagnosis of LVNC can be challenging due to the non-uniform diagnostic criteria. Echocardiography is the initial and basic tool for diagnosing this cardiomyopathy according to the morphological criteria [9,10]. So far, no separate morphological criteria for LVNC in children have been proposed. The most commonly used echocardiographic criteria are those provided by Jenni et al. [11].

In recent years, cardiovascular magnetic resonance (CMR) imaging has increasingly been used in the assessment of cardiomyopathies. It is currently considered the non-invasive gold standard for the evaluation of biventricular volumes, myocardial mass, regional and global systolic function, and tissue characteristics [12]. CMR may provide clinically relevant information and it allows for LVNC diagnosis, though the proposed diagnostic criteria vary. Because these criteria are based on small samples of patients and various assumptions, and because there is no accepted standard for children, their reliability remains undetermined for the pediatric population [13]. For CMR, Petersen’s criteria are most frequently used in clinical practice [14]. The emergence of CMR has enabled high-resolution imaging of cardiac structures, which provides detailed functional and morphologic information and allows for the presence and extent of fibrosis to be assessed [15]. Literature reports indicate that CMR is superior to echocardiography in assessing the extent of myocardial noncompaction, especially in areas which are not accessible by echocardiography, such as the left ventricular apex and the lateral wall [16]. 

The aim of our study was to evaluate echocardiographic and CMR findings in a group of children with isolated LVNC.

## 2. Material and Methods

### 2.1. Study Patients 

From February 2008 to July 2021, pediatric patients with echocardiographic features of LVNC who were hospitalized in the Department of Cardiology of the Children’s Memorial Health Institute were prospectively enrolled. The main reason for referring children to our reference cardiology center was suspicion of LVNC in echocardiography made in district centers and clinical symptoms, such as the following: heart failure, sinus bradycardia, cardiac arrhythmias, syncope, heart murmur, and family history of cardiomyopathy. The criteria for inclusion in the study were an age of less than 18 years at the time of diagnosis and echocardiographic evidence of isolated LVNC, defined as (1) the presence of a two-layer structure with a compacted and noncompacted endocardial layer of trabecular meshwork with deep endomyocardial spaces, (2) a maximal end-systolic ratio between the noncompacted (NC) and compacted (C) layers of 2.0 or greater, and (3) color Doppler evidence of deep perfused intertrabecular recesses. The exclusion criteria from the study were the presence of congenital heart disease, other forms of cardiomyopathy, or neuromuscular disorders. The institutional ethics committee approved this study. Informed consent was obtained from all individual participants included in the study.

### 2.2. Data Collection

Patients’ demographics, family history of cardiomyopathies and sudden cardiac death (SCD), and results from echocardiography, 12-lead resting electrocardiographic, 24-hour Holter electrocardiographic, and CMR were collected. NYHA/Ross functional class and clinical symptoms, such as chest pain, palpitations, syncope, pre-syncope, and thromboembolic events, were evaluated in all children. All children referred for CMR presented with echocardiographic features of LVNC and varying clinical symptoms, such as heart failure, cardiac arrhythmias, atrioventricular conduction disturbances, sinus bradycardia, syncope, chest pain, and a family history of cardiomyopathy and sudden cardiac deaths.

### 2.3. Echocardiographic Imaging and Analysis

Echocardiographic imaging was performed using a Philips Epiq7 (Philips Medical Systems, Bothell, WA, USA). Two-dimensional, Doppler, and M-mode echocardiography were performed at rest using standard methods. Echocardiographic images, including parasternal long- and short-axis and apical two-, three-, and four-chamber views were obtained and reviewed by cardiologists certified in echocardiography. 

Echocardiographic measurements were reviewed based on Jenni’s criteria [11] as follows: a ratio of NC) to C myocardial layer of 2.0 or greater, measured in the parasternal short-axis view in end-systolic phase below the papillary muscle. The NC/C ratio was additionally calculated in the parasternal short-axis projection in end-diastolic phase. Color Doppler imaging was performed in all children with visualization of the recess filling between the trabeculae with blood flowing in from the left ventricle (LV). LV dimension and systolic function were evaluated in detail. Echocardiographic measurements included LV end-diastolic (LVED) and end-systolic (LVES) volume [17] and area [18] in the apical four-chamber view, as well as LV diastolic and systolic diameters in the parasternal long-axis projection [19]. These parameters were evaluated for each patient and indexed to the patient’s BSA, according to Du Bois’ formula. Moreover, z-scores were calculated using the formula for z-scores reported in the literature [20]. LV systolic function was assessed by calculating the shortening fraction, ejection fraction (LV EF)—according to Simpson’s method—the value of mitral annulus peak systolic excursion (MAPSE) in mm, and z-score [21]. Left atrial dimension was measured at end-systole as the anteroposterior linear diameter from the parasternal long-axis view and was indexed to the patient’s BSA. The z-score for LAd was calculated with the formula for z-scores [17]. Left atrial enlargement was defined as a z-score greater than 2. It should be pointed out that the echocardiographic study also assessed the RV dimension and systolic function. RV diastolic diameter was evaluated in the parasternal long-axis view (mm, z-score) [17]. RV systolic function was assessed by calculating tricuspid annular plane systolic excursion values in mm and the z-score [22] and by measuring the fractional area change as a percentage of the difference between the RV end-diastolic and end-systolic areas evaluated in the apical four-chamber view.

### 2.4. CMR Imaging and Analysis

CMR imaging was performed using a 1.5-T scanner (Magnetom AvantoFit, Siemens, Erlangen, Germany), with a dedicated cardiac phased-array coil and electrocardiographic gating, as previously described [23]. Steady-state free precession (SSFP) cine images of the heart were acquired in the short-axis and four-, three-, and two-chamber planes with a minimum of 25 phases per cardiac cycle. Late gadolinium-enhanced (LGE) images were acquired in the short-axis and long-axis planes 10–15 min after intravenous administration of 0.1 mmol/kg of gadobutrol (Gadovist, Bayer, Berlin, Germany).

The studies were analyzed using CVi42 software (Circle Cardiovascular Imaging, Calgary, AB, Canada) on a dedicated diagnostic workstation. Cine images were used to determine the left and right ventricular volumes, ejection fraction, and left ventricular mass. The end-diastolic and end-systolic phases were identified based on long-axis and midventricular short-axis scans. The LV endocardial, epicardial borders, and the RV endocardial border were automatically contoured in those phases and then manually corrected to determine the end-diastolic (EDV) and end-systolic (ESV) volumes of both ventricles. Based on the results, the LV and RV stroke volumes (SV = EDV − ESV), and ejection fraction were calculated. Compacted LV mass, including the interventricular septum and the LV papillary muscles, was calculated based on segmentation in the end-diastolic phase. LV global mass was determined by manually drawing the LV endocardial border to include both papillary muscles and LV trabeculation. The LV noncompacted mass was then established by subtracting the compacted LV mass from the global LVM. As per the Petersen criteria, the thickness of the compacted and the noncompacted myocardial layers perpendicular to the compacted myocardium was measured in end-diastole in the three long-axis views (excluding the 17th segment according to the American Heart Association model) and the highest NC/C ratio value was recorded [14]. Additionally, analogously NC/C ratio was measured in diastole in the short-axis view in order to establish the number of segments with values > 2.3.

LV and RV compacted mass, EDV, ESV, SV, and LV trabeculation mass were indexed to the patient’s BSA, determined using Du Bois’ formula (BSA [m^2^] = 0.007184 × weight [kg]^0.425^ × height [cm]^0.725^). To identify morphological abnormalities, LV mass, LV EDV, and RV EDV were compared against recently published, multicenter, CMR normative values for children and adolescents, which were determined using the same methods [24]. Z-score values of less than −2.0 and greater than 2.0 were considered pathological.

The studies were visually assessed for the presence of myocardial LGE, which had to be present in two different spatial orientations. Additionally, the extent of LGE was quantitatively assessed using a dedicated module within CVi42, where pathological enhancement was defined as a myocardium with a signal intensity of more than 6 SD above the mean in a remote reference region of effectively nulled myocardium.

### 2.5. Statistical Analysis

The distribution of all continuous variables was assessed using the Shapiro–Wilk test. Normally distributed variables are presented as mean ± SD, whereas non-normally distributed parameters are given as median (interquartile range). Echocardiographic diagnostic performance was assessed in relation to CMR using standard accuracy criteria for binary diagnostic tests (i.e., sensitivity, specificity, and accuracy) with Clopper–Pearson confidence intervals and positive and negative predictive values with confidence intervals, calculated according to Mercado et al. [25]. Pearson’s correlation coefficient, and the Bland–Altman plot, were used to compare LV EDV between the imaging methods. Participants with myocardial LGE detected in CMR were compared with the children without myocardial LGE using an unpaired *t*-test or Mann–Whitney test, depending on the normality of the distribution. Categorical variables between groups were compared using the chi-squared test. *p*-values of less than 0.05 were considered statistically significant. Statistical analysis was carried out using MedCalc Statistical Software 20.014 (MedCalc Software Ltd., Ostend, Belgium).

## 3. Results

### 3.1. Clinical Characteristics 

A total of 34 patients with an echocardiographic diagnosis of LVNC were recruited between February 2008 and July 2021. The median age was 11.9 years (6.6–14.7) and 50% were male. The patients were followed prospectively for a median of 5.1 years (2.2–12.2). 

In the study group, 3% of patients were under one year of age; 32% were between 1 and 10 years of age; and 65% were over 10 years of age. Family history revealed cardiomyopathy in first-degree relatives in 11 children (32%) (LVNC in 20% of patients; both LVNC and DCM in 6%; LVNC and HCM in 3%; and HCM in 3%). Sudden cardiac deaths occurred in the families of three children (9%). The NYHA/Ross functional class in the majority of patients (74%) was evaluated as grade II; 3% had grade IV, while 24% had grade I. In 24-hour electrocardiographic Holter monitoring, the most prominent features were premature ventricular and atrial contractions, found in 26% and 15% of patients, respectively. Other findings were observed, including sinus bradycardia in 21% of children, paroxysmal third-degree atrioventricular block in 12%, ventricular tachycardia in 9%, and Wolff–Parkinson–White syndrome in 6% of patients.

### 3.2. Echocardiographic Results 

In 31 of the 34 patients (91%), the median NC/C ratio was 2.60 (IQR, 2.22, 3.40). In the remaining three patients (9%) referred from a regional center with a diagnosis of LVNC, the echocardiography performed in our cardiology center did not confirm the diagnosis, as the NC/C ratios ranged from 1.46 to 1.9. These patients were excluded from further analysis and were not referred for CMR examination.

CMR was performed in 27 of the 31 children (79%) who met Jenni’s criteria in echocardiography. In four (13%) patients, CMR was not performed due to their severe clinical condition and the implantation of an LV assist device for mechanical circulatory support (*n* = 1), an implanted pacemaker (*n* = 2), and hemodynamic instability and low body weight (*n* = 1). Among the 27 children who underwent CMR, the diagnosis of LVNC was confirmed in 25 (93%), according to Petersen’s criteria. In two patients (7%), the CMR investigations did not confirm echocardiographic diagnosis of LVNC, as the NC/C ratio was less than 2.3. 

A comprehensive and detailed analysis was performed on a group of 25 patients who met the echocardiographic and CMR criteria for LVNC diagnosis. The baseline characteristics of the study group are presented in Table 1.

In the group of 25 patients, the median NC/C ratio in systole was 2.60 (IQR, 2.22, 3.30) and in diastole 3.40 (IQR, 2.77, 4.80). In echocardiography, left ventricular systolic diameter was increased in 10 patients (40%) (LV diastolic diameter, 42–59.5 mm; z-score, +2.5 to +4.6). Of these, four patients (16%) had LV systolic function impairment (LV EF, 50%–55%; MAPSE, 9.6–16.5 mm; z-score, −2.8 to +1.6); in the remaining six children, LV EF was normal. No significant valvular abnormalities were noted in the study group. In two children, a reduction in LV EF was observed without an increase in LV diastolic diameter.

In one patient (4%), apart from LV enlargement and a reduction in LV EF, an impairment of RV systolic function was found (fractional area change, 30%; tricuspid annular plane systolic excursion, 18.5 mm; z-score, −1.5) with RV normal size. On the other hand, in one patient (4%), RV enlargement (36 mm; z-score, +2.8) with normal systolic function was observed. Left atrial enlargement was found in two patients (8%) (LAd, 35 mm; z-score, from +3.4 to +4). Table 2 presents the results of echocardiographic and CMR imaging from 25 patients with LVNC.

### 3.3. CMR Results

**Figure 1:**
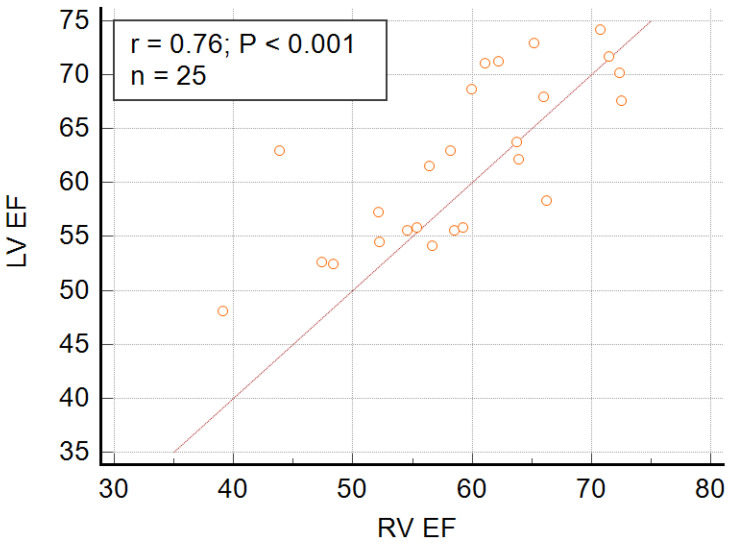
Correlation between RVEF and LVEF.

Twenty-seven participants meeting the echocardiographic criteria of LVNC underwent CMR with LGE assessment. In 25 out of 27 children (93%), LVNC was confirmed by CMR, according to Petersen’s criteria, with a median NC/C ratio of 3.27 (IQR, 2.56,3.76) and on average 5.1 ± 1.5 noncompacted segments. LV enlargement was diagnosed in 5 out of 25 children (20%) with LVNC, LV function impairment was diagnosed in 6 of the 25 (24%) patients, RV enlargement in four (16%), RV function impairment in seven (28%), and left atrial enlargement in five (20%) (Table 2). RVEF was strongly correlated with LVEF (*r* = 0.76; *p* < 0.001) (Figure 1), though it was not associated with LV or RV volumes.

**Figure 2:**
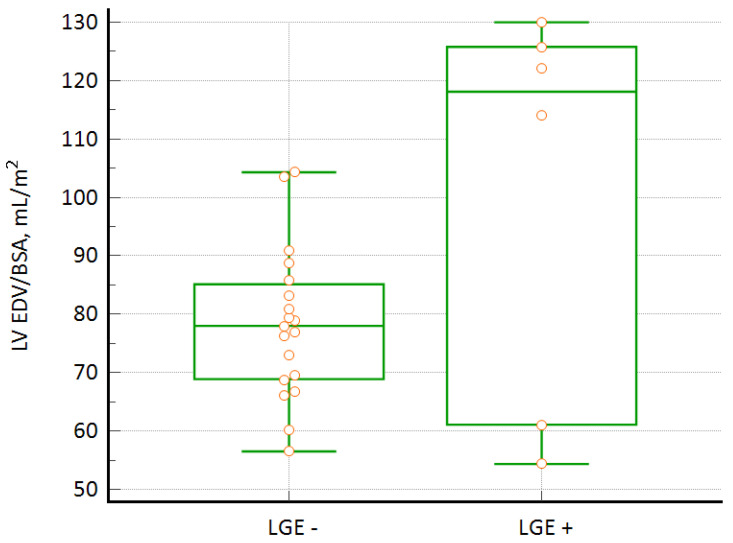
LV EDV/BSA comparison between LVNC patients with and without LGE.

In 6 out of the 25 patients (24%), midwall LGE was observed involving on average 6.6% ± 2.4% of the LV myocardial mass. In all of the patients, LGE was observed in at least one basal segment, and anterior segments (according to the AHA model) were most commonly involved (in four out of the six patients with LGE, 67%). LGE was noted in both, compacted and noncompacted segments. Compared to the children with LVNC without LGE, they had larger LV, characterized by higher LV EDV/BSA (101 ± 34 vs. 78 ± 13 mL/m^2^, *p* = 0.02) (Figure 2), as measured by CMR, and higher LV EDV index, as measured in echocardiography (61 ± 13 vs. 77 ± 19 mL/m^2^; *p* = 0.032). They also had higher LV SV/BSA (62 ± 21 vs. 49 ± 9 mL/m^2^; *p* = 0.026), RV SV/BSA (63 ± 22 vs. 48 ± 9 mL/m^2^; *p* = 0.022), and LV compacted mass/BSA (63 [48, 70] vs. 48 [44, 53] g/m^2^; *p* = 0.043). The groups of LVNC patients with and without LGE did not differ in the other parameters assessed in CMR or echocardiography, including NC/C ratio, LV EF, RV EF, RV EDV/BSA, or left atrial maximum volume.

### 3.4. Comparison of Echocardiographic and CMR Results 

In the CMR investigations, NC/C ratio significantly correlated with echocardiographic NC/C ratio measured in systole (*r* = 0.41; *p* = 0.044), but not in diastole. 

There were no significant correlations between NC/C and LV volumes or function. When referenced to CMR, which is considered as the gold standard for ventricular size and function assessment, echocardiographic examination had high accuracy for detecting LV function impairment (92% (74–99%)), with high specificity (95% (74–100%)), and moderate sensitivity in children with LVNC.

However, the LV EF values measured using the two imaging methods were not significantly correlated (*r* = 0.36; *p* = 0.08). The mean difference between the echocardiographic and CMR results was −2.4% ± 7.8% and the lower and upper limits of agreement (LoA) were −18.1% and 13.3%, respectively.

**Figure 3:**
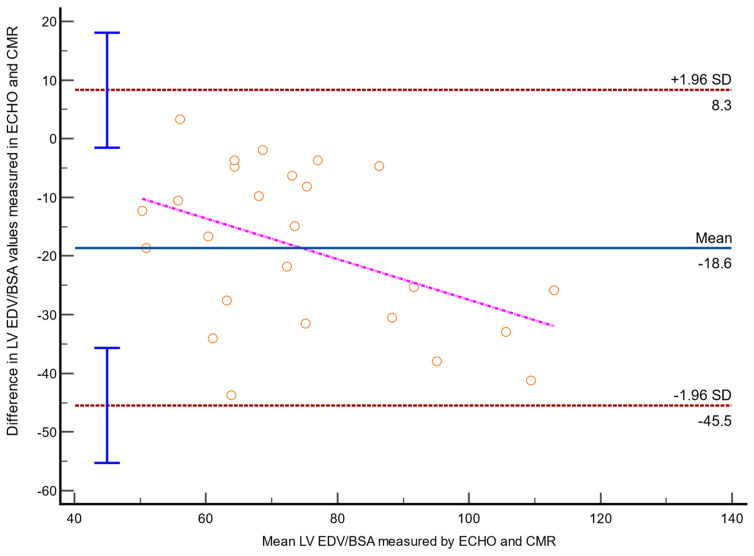
Bland–Altman plot of left ventricular end-diastolic volume mass indexed to body surface area (LV EDV/BSA). ECHO indicates echocardiography; CMR, cardiovascular resonance imaging; SD, standard deviation.

Echocardiography had moderate sensitivity for diagnosing LV enlargement (80% (28–99%)), but its specificity and overall accuracy in this aspect were relatively low (65% (41–85%)) and 68% (47–85%), respectively). Nevertheless, the results acquired with these methods were very strongly correlated (*r* = 0.93; *p* < 0.0001). The mean difference between the echocardiographic and CMR LV EDV results was −25.9 mL (lower and upper LoA, −67 mL and 15.2 mL, respectively); the mean difference between LV EDV/BSA measurements was −18.6 mL/m^2^ (lower and upper LoA, −45.5 mL/m^2^ and 8.3 mL/m^2^, respectively) (Figure 3). 

For both absolute and indexed LV EDV, the differences increased with higher values (regression coefficients, −0.29; *p* = 0.001 and −0.35; *p* = 0.02, respectively).

Echocardiography had high specificity for detecting RV function impairment (100% (81–100%)) and enlargement (95% (76–100%)); however, its sensitivity in the assessment of those abnormalities was very low (14% (0–58%) and 25% (1–81%), respectively). Similarly, echocardiography had very high specificity in left atrial enlargement diagnosis (100% (83–100%)), but its sensitivity in this aspect was relatively low (40% (5–85%)).

## 4. Discussion

The main findings of this prospective observational study on LVNC in children are as follows:

1. Almost one fourth of pediatric patients with LVNC present with features of myocardial fibrosis;
2. Right ventricular abnormalities, which are often present in children with LVNC, can only be reliably assessed with CMR.

Among the cardiac imaging techniques used in patients with LVNC, echocardiography and CMR are the primary diagnostic methods. The advantages of echocardiography over CMR are that it is more available, the costs of examination are lower, and there is no need for anesthesia in younger children. Consequently, echocardiography is the first choice in the diagnosis of LVNC [26]. Echocardiography, however, has its limitations. First of all, there is a wide range of echocardiographic diagnostic criteria in the literature, based on studies with small samples using different research methodologies [9,17]. The cardiac cycle (end-systole or end-diastole), in which the measurements of the noncompacted and compacted layers are made, is also important as the thickness of the myocardium is maximal in systole and minimal in diastole, which directly affects the NC/C ratio. The next point of discussion is the echocardiographic projection, in which the measurements for the NC/C ratio should be made. Most of the published diagnostic criteria suggest that these measurements should be performed in the LV parasternal short-axis view; however, the apical four- and two-chamber views are most commonly used in everyday clinical practice. Finally, there is no uniform consensus on the threshold value of the NC/C ratio to use as a diagnostic criterion for LVNC [17]. The most frequently used criteria are those presented by Jenni et al., which are dedicated to adult patients; the suggested NC/C ratio is 2:1 or higher [11]. These echocardiographic criteria were used in our study, as in other published studies on children with LVNC [27], although some authors have proposed an NC/C ratio of greater than 1.4 as diagnostic criterion for LVNC in the pediatric population [28].

Improvements in cardiac imaging modalities, such as echocardiography and CMR imaging, have increased the identification of LVNC [29]. CMR is superior to echocardiography methodologies with regard to the number of segments that can be analyzed and the evaluation of the extent of two-layered myocardia. Moreover, CMR imaging has the potential to detect segmental non-compaction in any area of the LV wall and can provide supplemental morphological information beyond that obtained from conventional echocardiography [30]. Only a few previous studies have compared NC/C ratios assessed by CMR versus echocardiography [30,31]. The advantage of our study is that, for the first time, it compares data obtained with CMR and standard echocardiography in a larger group of pediatric patients. The results of our study demonstrate that in as many as 93% of children with LVNC features on echocardiography, CMR confirmed the diagnosis of the disease, which indicates that echocardiography is a precise diagnostic method for LVNC assessment in children.

Some authors have emphasized the role of better visualization of the noncompacted layer of the myocardium and trabeculae in end-diastole in echocardiography [32], while others have shown that end-systolic measurements of LVNC in CMR have stronger associations with cardiac events [33]. In our pediatric study, echocardiography images obtained at end-systole and end-diastole were compared with those obtained by CMR at end-diastole to assess NC/C ratio. Only systolic—not diastolic—NC/C ratios measured in echocardiography significantly correlated with NC/C ratio measurements in CMR, which is different from the results of a study on adult patients that reported good agreement between echocardiography at end-diastole and CMR measurements [30]. The results of our study suggest a strong advantage of evaluating the noncompacted myocardium during systole in echocardiographic studies. 

Other authors [34,35] have assessed the correlation between NC/C ratio and LV EF. The results of these studies revealed that patients with increasing severity of noncompaction in echocardiography had significantly lower LV EF and LV EF correlated with parameters of specific diagnostic criteria for LVNC in CMR, such as an NC/C ratio greater than 2.3 and a more than 20% proportion of the noncompacted myocardium being LV mass. We did not find such a correlation in our study group. Nevertheless, 24% of the participants with LVNC confirmed by CMR presented with LV systolic function impairment, which is an important finding, as decreased LV EF is a significant risk factor [36]. Moreover, we observed a high accuracy of echocardiography in diagnosing LV systolic function impairment when referenced to the CMR, indicating its utility in patient follow-up. LV enlargement was observed in CMR in 5 of the 25 participants with LVNC (20%), indicating a significant incidence of LV remodeling in children with LVNC. Admittedly, echocardiography had moderate sensitivity for diagnosing LV enlargement (80%), though its specificity and overall accuracy in this aspect was relatively low. 

Contrast-enhanced CMR with LGE imaging may detect myocardial fibrosis [37]. It is relatively frequently observed in patients with LVNC, though its presence or absence is not a reliable diagnostic marker of the disease [38]. In a study by Grothoff et al. [39], none of the LVNC patients demonstrated LGE, while other authors have described the presence of LGE in isolated LVNC, which is associated with a poorer LV systolic function [40,41]. In our study, we found LGE in pediatric patients with isolated LVNC and confirmed a relationship between LGE and features of LV remodeling. As in the case of other authors [42], in our study group the presence of LGE was associated with higher values of LVEDV. In contrast, LV EF did not differ between LVNC patients with LGE and other children with LVNC, similar to a study by Andreini et al. [32]. As the presence of LGE in adult LVNC patients was shown to be a significant risk factor [36] of cardiovascular events, our findings of significant incidence of myocardial fibrosis in children with LVNC and associated LV remodeling indicate the clinical importance of CMR imaging in the routine evaluation of those patients [32], as in children with hypertrophic cardiomyopathy [43]. 

The results of studies published in the literature [28] indicate a significant share of RV systolic dysfunction in patients with isolated LVNC. There are reports [44] that have emphasized the relationship between RV systolic dysfunction and significantly lower LV EF and relevant LV enlargement. According to the authors [28], patients with impaired RV systolic function have a greater LV volume, lower LV systolic function, and more pronounced myocardial fibrosis, which may indicate that RV dysfunction is a marker of a more advanced stage of LVNC. The results of our study showed a significant correlation between left and right ventricular function, but we did not prove a relationship between RV EF and the size of the right and left ventricles. The significant incidence of RV enlargement and RV systolic function impairment observed in children with LVNC further highlights the clinical significance of CMR imaging in this population, since the possibilities of echocardiography RV evaluation are limited.

Based on our experience, we can summarize that echocardiography should be used as the first diagnostic test in LVNC, while CMR is strongly recommended as a complementary examination to accurately assess the extent of myocardial noncompaction and to reliably analyze the size and systolic function of the ventricles.

The results of studies on LVNC in children published so far require further research due to the many unanswered questions regarding diagnostic methods, diagnosis, and clinical management. 

## 5. Conclusions

1. In the morphological assessment of myocardial noncompaction among the study group of pediatric patients, there was a very good agreement between echocardiography and CMR imaging;
2. A significant correlation was demonstrated in the assessment of NC/C ratio from end-systole measurements in echocardiography and in end-diastole measurements in CMR examination;
3. Echocardiography is a good method for monitoring LV systolic function, but CMR is indicated for precise assessment of the left ventricular morphology and enlargement;
4. CMR significantly exceeds echocardiography in the assessment of the right ventricle in children with LVNC and should be included in the basic diagnostics of these patients;
5. CMR imaging allows for the detection of areas of LGE, which are indicative of myocardial fibrosis;
6. LGE incidence is relatively high in pediatric patients with LVNC and is associated with LV remodeling. As it is also a risk factor of future cardiovascular events, contrast-enhanced CMR should be a part of a standard diagnostic work-up of pediatric patients with LVNC.

## Figures and Tables

**Table 1:** Baseline characteristics (*n* = 25).

Sex (% Male)	44% (11/25 Boys)
Age (years)	11.3 ± 4.0
Height (cm)	144 ± 25
Weight (kg)	39 ± 19
Body mass index (kg/m^2^)	17.5 ± 3.8
Body surface area (m^2^)	1.25 ± 0.41
Heart rate (beats per minute)	73 ± 20

**Table 2:** Echocardiographic and CMR imaging studies.

	All Patients (*n* = 25)
**Echocardiography**	
NC in systole, mm, median, IQR	11.0 (9.8, 15.8)
C in systole, mm, median, IQR	4.4 (3.7, 4.8)
NC/C ratio in systole, median, IQR	2.60 (2.22, 3.30)
NC in diastole, mm, mean, SD	13.2 ± 4.5
C in diastole, mm, mean, SD	3.6 ± 1.3
NC/C ratio in diastole, median, IQR	3.40 (2.77, 4.80)
LVED vol., mL, median (IQR)	79 (50, 100)
LVED vol. index, mL/BSA, mean, SD	65 ± 16
LVES vol., mL, mean, SD	34 ± 16
LVES vol. index, mL/BSA, mean, SD	27 ± 8
LVED area, cm^2^, mean, SD	25 ± 8
LVED area z-score, mean, SD	0.74 ± 1.46
LVES area, cm^2^, mean, SD	14 ± 5
LVES area z-score, mean, SD	−1.04 ± 1.76
LV diastolic diameter, mm, median, IQR	45 (37, 52)
LV diastolic diameter z-score, mean, SD	1.39 ± 1.68
LV systolic diameter, mm, median, IQR	30 (25, 36)
LV systolic diameter z-score, median, IQR	1.30 (0.00, 3.15)
LV EF acc. Simpson formula, %, mean, SD	60 ± 6
Shortening fraction, %, mean, SD	34 ± 5
MAPSE lateral, mm, mean, SD	14 ± 3
MAPSE lateral z-score, mean, SD	−0.60 ± 2.24
Left atrial diameter, mm, mean, SD	28 ± 4
Left atrial diameter z-score, mean, SD	0.65 ± 1.38
RV diastolic diameter, mm, mean, SD	21 ± 5
RV diastolic diameter z-score, median, IQR	0.25 (−0.10, 0.80)
TAPSE, mm, mean, SD	20 ± 7
TAPSE z-score, mean, SD	−0.29 ± 2.95
RV FAC, %, mean, SD	45 ± 7
LV enlargement (LV diastolic diameter z-score > 2.0)	10/25 (40%)
LV function impairment (LV EF ≤ 55%)	6/25 (24%)
RV enlargement (RV diastolic diameter z-score > 2.0)	1/25 (4%)
RV function impairment (FAC ≤ 35%)	1/25 (4%)
Left atrial enlargement (LAd z-score > 2.0)	2/25 (8%)
**Cardiovascular magnetic resonance**	
LV EDV, mL, mean, SD	107 ± 51
LV EDV/BSA mL/m^2^, mean, SD	84 ± 22
LV EDV, z-score, mean, SD	0.65 ± 1.61
LV ESV, mL, median, IQR	33 (24, 52)
LV ESV/BSA, mL/m^2^, median, IQR	32 (22, 37)
LV ESV, z-score, mean, SD	1.24 ± 1.43
LV SV, mL, mean, SD	66 ± 30
LV SV/BSA, mL/m^2^, mean, SD	52 ± 14
LV SV, z-score, mean, SD	0.70 ± 1.64
LV compacted mass, g, mean SD	68 ± 27
LV compacted mass/BSA, g/m^2^, median, IQR	50 (45, 58)
LV compacted mass, z-score, median, IQR	−0.18 (−0.71, 0.47)
LV trabeculated mass, g, median IQR	24 (21,34)
LV trabeculated mass/BSA, g/m^2^, median IQR	21 (16, 28)
LV trabeculated/nontrabeculated mass, %, median, SD	29 ± 7
LV EF, %, mean, SD	62 ± 8
LV CI, L/m^2^, mean, SD	3.62 ± 0.76
RV EDV, mL, mean, SD	111 ± 51
RV EDV/BSA, mL/m^2^, mean, SD	87 ± 21
RV EDV, z-score, mean, SD	0.44 ± 1.52
RV ESV, mL, mean, SD	46 ± 25
RV ESV, mL/m^2^, mean, SD	36 ± 12
RV ESV, z-score, mean, SD	0.57 ± 1.67
RV SV, mL, mean, SD	65 ± 30
RV SV/BSA, mL/m^2^, mean, SD	51 ± 14
RV SV, z-score, mean, SD	0.18 ± 1.46
RV EF, %, mean, SD	59 ± 9
RV EF, z-score, mean, SD	−0.87 ± 2.18
RV CI, L/m^2^, median, IQR	3.45 (3.20, 3.75)
LV NC, mm, median, IQR	12.5 (11.8, 13.7)
LV C, mm, mean, SD	4.0 ± 0.7
LV NC/C, median, IQR	3.27 (2.56, 3.76)
Number of noncompacted segments	5.1 ± 1.5
Left atrial max volume, mL, mean, SD	43 ± 19
Left atrial max volume/BSA, mL/m^2^, median, IQR	30 (27, 43)
LV enlargement (LV EDV z-score > 1.65)	5/25 (20%)
LV function impairment (LV EF < 55%)	6/25 (24%)
RV enlargement (RV EDV z-score > 1.65)	4/25 (16%)
RV function impairment (RV EF < 55%)	7/25 (28%)
Left atrial enlargement (>97 percentile)	5/25 (20%)

NC—noncompaction of ventricular myocardium, C—compaction of ventricular myocardium, LVED vol.—left ventricular (LV) end-diastolic volume, LVES vol.—left ventricular end-systolic volume, LV EF—left ventricular ejection fraction, MAPSE—mitral annulus peak systolic excursion, LAd—left atrial diameter, TAPSE—tricuspid annular plane systolic excursion, RV FAC—right ventricular fractional area change.

## Data Availability

The data presented in this study are available on request from the corresponding author.

## Abbreviations

BSA	Body surface area
C	Compacted myocardial layer
CMR	Cardiovascular magnetic resonance
EF	Ejection fraction
FAC	Fractional area change
LGE	Late gadolinium enhancement
LoA	Limits of agreement
LV	Left ventricle
EDV	End-diastolic volume
ESV	End-systolic volume
LVNC	Left ventricular noncompaction
MAPSE	Mitral annulus peak systolic excursion
NC	Noncompacted myocardial layer
NTproBNP	N-terminal pro-brain natriuretic peptide
RV	Right ventricle
SCD	Sudden cardiac death
SD	Standard deviation
SV	Stroke volumes
TAPSE	Tricuspid annular plane systolic excursion
